# United States Veterinary Medical Association

**Published:** 1890-05

**Authors:** 


### EDITORIAL.

### UNITED STATES VETERINARY MEDICAL ASSOCIATION.

WE again bring before all the members of the United States Veterinary Medical Association a reminder of the date of the annual meeting at Chicago, September 16th and 17th, 1890.

This meeting is to establish a new era, in which we are no longer to have a one-day's meeting, occupied almost entirely by business, with no time for proper discussion at the end of the day. At this meeting we are to get through with the business promptly, and are to have several papers of importance.

Dr. D. E. Salmon, chief of the Bureau of Animal Industry, Washington, D. C., will present a paper on "The Last Studies in Bacteriology as it Refers to the Domesticated Animals and the Diseases in Them." As there is no one better qualified to present this subject at a medical meeting—as the ferments are causes of disease now forming one of the most important elements in the study of medicine—this paper will be of great value.

Prof. A. Liautard, Dean of the American Veterinary College, will address the association. We have not yet been informed of the subject which he will present, but his reputation for clever and valuable papers assures us an interesting and profitable hour.

Dr. R. S. Huidekoper will present a practical paper, with illustrations, on "Contraction of the Horse's Foot and Contracted Heels," especially entering into the rectification of the deformities of the foot by proper shoeing. Other addresses will undoubtedly be given.

The Committee of Arrangements propose to organize a party, which shall collect from the New England States at New York, and leave there Sunday, September 14th, pass through Philadelphia and Baltimore, taking up, on the way, the members of the association in the neighborhood, and proceed, *via* Cincinnati, to Lexington, Kentucky, where they will arrive early Monday morning.

The party will pass one day at Lexington, Ky., where arrangements will be made for them to visit the most important stock farms and see some of the most valuable stallions and brood mares in the United States; they will leave that night and arrive in Chicago in time for the first day's meeting.

The party will return, after the second day's meeting, directly to New York. The exact price of the round trip, including the Pul man sleeper, has not yet been obtained, but the committee are promised a very great reduction from ordinary rates. A reduction in rates will also be obtained from Boston, New York and Philadelphia, direct to Chicago and return, for those who are not able to join the first party.

The committee desire to hear at once from each member of the association, and learn from him if he will form one of the party, as, when they know the approximate number, they can make more definite and better terms as to cost.

Will each member please write at once to Dr. W. H. Hoskins, chairman of the Committee of Arrangements, 12 S. Thirty-seventh Street, Philadelphia, in answer to the above?

We beg to urge upon every Eastern member of the association the importance of attending this meeting, and to remind them that in making their Summer's plans, that they should set aside at least five days, from the 14th to the 19th of September, to join their colleagues in a trip to and from Chicago, and be present at the meeting, where they will have the opportunity of meeting á large number of Western veterinarians and future members of the association.

### PAPER HORSESHOES.

A SERIES of experiments are being made in Germany to manufacture horseshoes from a material into which paper enters for the most part. This shoe adheres to the horse's foot better than a metal one. It is impervious to the action of water, and as use makes it rough, it prevents the horse from slipping.—*Revue Scientifique.*

